# Detection of 4-formylaminooxyvinylglycine in culture filtrates of *Pseudomonas fluorescens* WH6 and *Pantoea ananatis* BRT175 by laser ablation electrospray ionization-mass spectrometry

**DOI:** 10.1371/journal.pone.0200481

**Published:** 2018-07-10

**Authors:** Rachel A. Okrent, Kristin M. Trippe, Viola A. Manning, Callee M. Walsh

**Affiliations:** 1 USDA-ARS Forage Seed Production Research Unit, Corvallis, Oregon, United States of America; 2 Department of Crop and Soil Sciences, Oregon State University, Corvallis, Oregon, United States of America; 3 Protea Biosciences, Inc., Morgantown, West Virginia, United States of America; Tallinn University of Technology, ESTONIA

## Abstract

The oxyvinylglycine 4-formylaminooxyvinylglycine (FVG) arrests the germination of weedy grasses and inhibits the growth of the bacterial plant pathogen *Erwinia amylovora*. Both biological and analytical methods have previously been used to detect the presence of FVG in crude and extracted culture filtrates of several *Pseudomonas fluorescens* strains. Although a combination of these techniques is adequate to detect FVG, none is amenable to high-throughput analysis. Likewise, filtrates often contain complex metabolite mixtures that prevent the detection of FVG using established chromatographic techniques. Here, we report the development of a new method that directly detects FVG in crude filtrates using laser ablation electrospray ionization-mass spectrometry (LAESI-MS). This approach overcomes limitations with our existing methodology and allows for the rapid analysis of complex crude culture filtrates. To validate the utility of the LAESI-MS method, we examined crude filtrates from *Pantoea ananatis* BRT175 and found that this strain also produces FVG. These findings are consistent with the antimicrobial activity of *P*. *ananatis* BRT175 and indicate that the spectrum of bacteria that produce FVG stretches beyond rhizosphere-associated *Pseudomonas fluorescens*.

## Introduction

Oxyvinylglycines are a small class of biologically-active molecules produced by certain strains of bacteria [1]. Examples of oxyvinylglycines include aminoethoxyvinylglycine from *Streptomyces sp*. [2], 4-methoxyvinylglycine from *Pseudomonas aeruginosa* [3], rhizobitoxine from *Bradyrhizobium elkanii* [4], and 4-formylaminooxyvinylglycine (FVG) from *P*. *fluorescens* [5]. FVG is a potent secondary metabolite that arrests the germination of weedy grasses [6] and demonstrates antibiotic activity against the bacterial plant pathogen *Erwinia amylovora* [7].

The lability and hydrophilicity of oxyvinylglycines complicate efforts to precisely detect FVG or FVG-like compounds in crude culture filtrates. The routine determination of an FVG phenotype therefore requires an ethanol-based extraction of crude culture filtrate [8]. The presence or absence of FVG is subsequently determined by simultaneously examining partially purified filtrates with thin-layer chromatography [9], and two biological assays [6,7]. Simultaneous analyses are required because each method in itself cannot unequivocally identify FVG as the active molecule. For example, we have observed that thin-layer chromatograms of other compounds are remarkably similar to thin-layer chromatograms of FVG (data not shown). In addition, the biological activities of other vinylglycines, including aminoethoxyvinylglycine and 4-methoxyvinylglycine, overlap significantly with FVG [7,10]. Collectively, the extraction and analysis of crude culture filtrate using current methodologies is time-consuming and requires large sample volumes. Because of these challenges, we have sought a high-throughput and precise method to directly detect FVG in crude culture filtrates.

Laser ablation electrospray ionization (LAESI) is a sample introduction technique for mass spectrometry. In the LAESI process, a mid-infrared laser ablates samples to introduce molecules into the gas phase, which then collide with an intersecting electrospray, leading to ionization and detection by a mass spectrometer [11]. LAESI-MS is suitable for analyzing small volumes of aqueous samples, is tolerant to salts, and is very rapid (15 s or less per sample). It can be used for high-throughput screening of complex, native biological samples, as has been demonstrated recently for detection of domoic acid toxin in shellfish homogenates [12]. LAESI-MS has been successfully used to detect metabolites from bacterial colonies on an agar surface [13,14] and in a biofilm [15], but has not yet been reported for detecting metabolites in crude culture filtrate.

**Fig 1:**
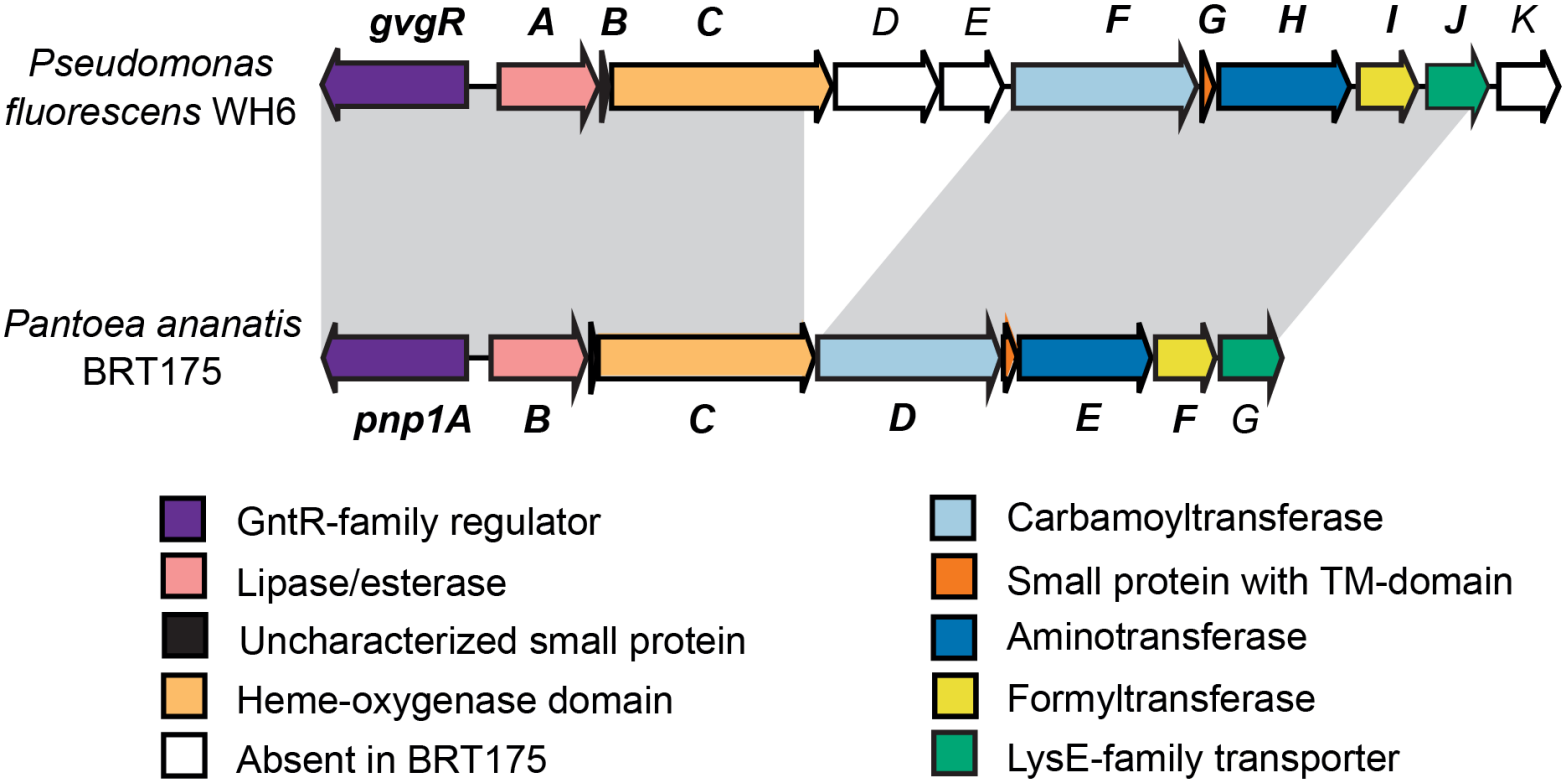
Comparison of the *gvg* and PNP-1 clusters from *Pseudomonas fluorescens* WH6 and *Pantoea ananatis* BRT175. Gene arrows are colored based on the class of the encoded protein. Gene designations in bold indicate genes that, when mutated, result in a null-FVG phenotype and/or antibiotic activity against *Erwinia amylovora*. Gray shading indicates homologous regions of the *gvg* cluster in each strain. TM, transmembrane.

To date, FVG has only been detected in strains of *P*. *fluorescens* [5]. However, additional bacterial strains are predicted to produce FVG or FVG-like compounds. *Pantoea ananatis* BRT175 produces a compound that, like *P*. *fluorescens* WH6, has activity against *E*. *amylovora*. This compound is linked with a biosynthetic gene cluster similar to one associated with FVG production in WH6 (Fig 1) [16–19]. The aims of the current study were to: 1) develop methods for LAESI-MS-based detection of FVG directly in bacterial culture filtrates, and, 2) validate this method by directly analyzing culture filtrates from BRT175 to determine if FVG is produced by this non-pseudomonad.

## Materials and methods

### Preparation of culture filtrates

Bacterial strains are listed in Table 1. *Pseudomonas fluorescens* and *Pantoea ananatis* strains were inoculated into either 60 ml or 5 ml of Pseudomonas Minimal Salts Medium (PMS) [6] in a 125-ml Wheaton bottle or 20-ml test tube, respectively. The cultures were grown at 28 °C with shaking for either seven (60-ml cultures) or two days (5-ml cultures). Subsequently, cells were removed from the filtrate by centrifugation (3000 x g for 15 min) and filter sterilized (Millipore GP express Steritop, 0.22-μm pore size (EMD Millipore, Billerica, MA, USA) or 25-mm Pall Acrodisc with 0.22-μm pore size Supor membrane (Pall Corporation, Port Washington, NY, USA), respectively). The dilution series was prepared by concentrating the culture filtrate in a Savant SPD1010 SpeedVac Concentrator (Thermo Scientific, Waltham, MA, USA) without heat (2X), or by diluting (0.3X, 0.1X, 0.03X, 0.01X) culture filtrate with PMS media. As a positive control, a sample enriched in FVG was prepared by extraction with 90% ethanol as described in [9].

**Table 1:** Strains used in this study.

Strains	Relevant Characteristics	Reference
*Pseudomonas fluorescens*		
WH6	Wild type; from *Triticum aestivum* L. roots; Ap^r^	[20]
WH6-28G	Δ*gvgF*; carbamoyltransferase-encoding gene deleted; Ap^r^	[16]
WH6-30G	Δ*gvgH*; aminotransferase-encoding gene deleted; Ap^r^	[16]
WH6-31G	Δ*gvgI*; formyltransferase-encoding gene deleted; Ap^r^	[16]
*Pantoea ananatis*		
BRT175	Wild type; from strawberry	[18]
BRT175-*pnp1D*::Tn*5*	Contains Tn*5* insertion in *pnp1D*, (carbamoyltransferase-encoding gene); Km^r^	[18]
*Erwinia amylovora* 153	Wild type; from a fire blight canker on Gala apple	[21]

Ap^r^, ampicillin resistant; Km^r^, kanamycin resistant.

### Biological assays and thin layer chromatography

The agar diffusion bioassay for anti-microbial activity against *E*. *amylovora* was performed as previously described [7]. The areas of the zones of inhibition were measured on triplicate plates for each sample using Able Image Analyser (Mu Labs). Culture filtrates were extracted with 85% ethanol and analyzed by TLC as described in [9]. The semi-quantitative bioassay for germination arrest activity was performed using the standard protocol and scoring system described in [6] for annual bluegrass (*Poa annua* L.) seeds. Briefly, each well of a 48-well culture plate (Corning Costar 3548) received a 200-uL aliquot of extracted or crude culture filtrate and three surface- sterilized annual bluegrass seeds. For each treatment, three replicate wells were prepared. The plates were sealed with parafilm and incubated for 7 d at 20°C (8 h light, 16 h dark d^-1^). Seed germination was subsequently scored using the system described by Banowetz [6]. A score between 0 and 4 is assigned to each seed, and nine seeds per treatment are scored. A score of 0 indicates that no visible signs germination are evident under 10X magnification. A score of 4 indicates that the first true leaf is longer than the coleoptile and that roots have elongated. The details of the scoring system between 1 and 4 are described in S1 Fig.

### LAESI-MS analysis

LAESI-MS analysis was performed by Protea Biosciences, Inc. Samples of crude culture filtrate, non-inoculated filtrate, or extracted culture filtrate (20 μl) were aliquoted into individual wells of a 96-well plate and analyzed with a LAESI DP-1000 (Protea Biosciences, Morgantown, WV, USA) coupled to a Q Exactive Orbitrap mass spectrometer (Thermo Scientific). In the initial experiments with extracted and crude culture filtrate from WH6 and mutant strains, settings for the LAESI DP-1000 were electrospray (+ 4000 V, 1.25 μl/min flow, 50% methanol, 0.1% acetic acid), 20 pulses per well at 1 Hz laser repetition rate, and laser energy 1000 μJ. A full scan from *m/z* 50 to 750 was performed for detection of FVG [M+H]^+^ *m/z* 161.0556 and [M+Na]^+^ *m/z* 183.0374 with an error of 1.1 ppm. MS/MS was performed on the precursor ion *m/z* 183.037 [M+Na]^+^ of the sodium adduct of FVG using a Q Exactive Orbitrap mass spectrometer. Conditions were slightly altered for analysis of a dilution series of culture filtrates from WH6 and the comparison of BRT175 to WH6 samples. The conditions of ablation and scanning were optimized for WH6 culture filtrate and a media-only control. In subsequent runs, settings for the LAESI DP-1000 were electrospray (+ 4000 V, 1.0 μl/min flow, 50% methanol, 0.1% acetic acid), 100 pulses per well at 20 Hz laser repetition rate, and laser energy ~600 μJ. The samples were analyzed in selected-ion monitoring mode at a resolution of 140,000 with scans centered on the sodium adduct of FVG, [M+Na]^+^ *m/z* 183.037. The average scan signal intensities were extracted on each well using LAESI Bridge software. Measurements were also performed on a second set of biological replicates for comparison.

## Results and discussion

### FVG-null mutant strains

The genetic basis of FVG production has previously been investigated in *P*. *fluorescens* WH6 [17,22]. Recently, Okrent et al. comprehensively examined the importance of each gene within the *gvg* biosynthetic gene cluster [16], which encodes proteins with regulatory, biosynthetic and transport functions (Fig 1). In that study, deletions in the biosynthetic genes encoding a carbamoyltransferase (*gvgF)*, aminotransferase (*gvgH*) and formyltransferase (*gvgI)*, led to loss of FVG production [16]. These mutant strains were used as controls in the development of methods for LAESI-MS-based detection of FVG in the current study.

### Feasibility of LAESI-MS for detection of FVG

In initial trials of LAESI-MS, the sodium adduct of FVG (*m/z* 183.0372 with 1.1 ppm mass error) was detected in *P*. *fluorescens* WH6 culture filtrates that had been extracted with 90% ethanol. Fragmentation of the precursor ion by MS/MS resulted in characteristic fragments similar to those previously obtained from a low resolution MS/MS of the *m/z* 183 [M+Na]^+^ peak (S2 Fig) [5].

**Fig 2:**
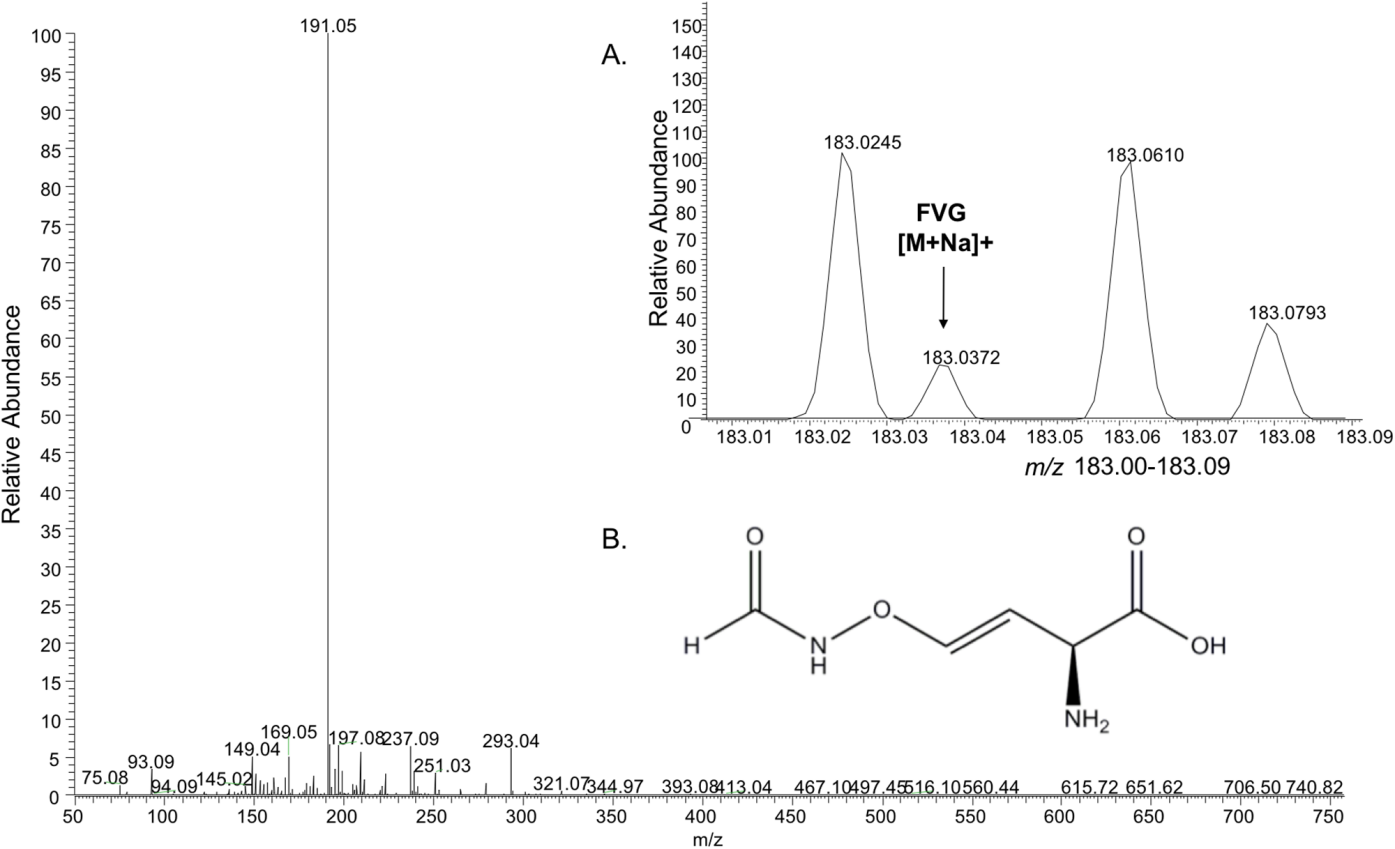
Laser ablation electrospray ionization-mass spectrometry analysis of crude culture filtrate from wild-type *Pseudomonas fluorescens* WH6. An MS spectrum from *m/z* 50 to 750 is shown with inset (A.) showing the peaks within the range *m/z* 183.00–183.09 only. The chemical structure of 4-formylaminooxyvinylglycine (FVG) is shown in inset B.

**Fig 3:**
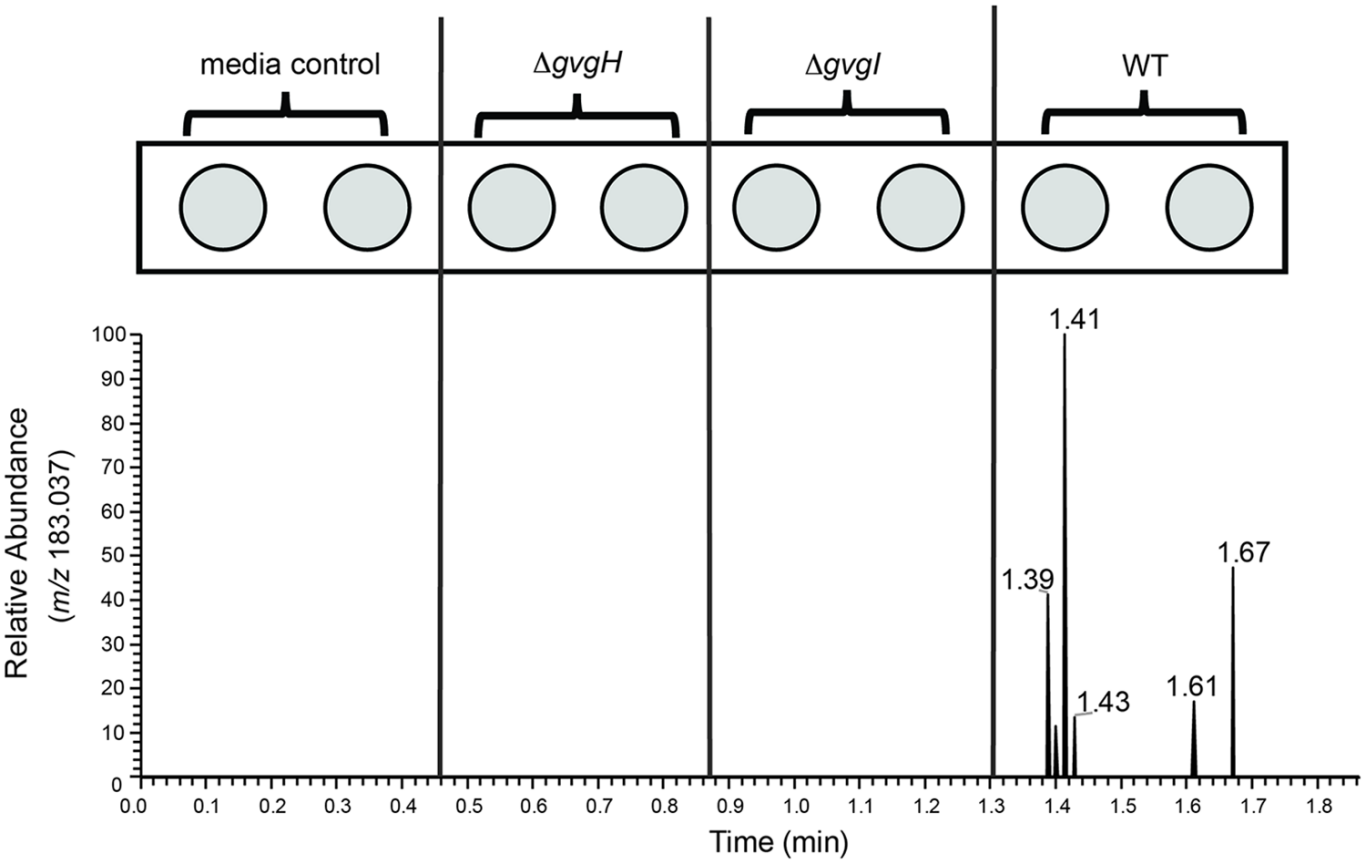
A scan for FVG ions in crude filtrate from WT and null-FVG mutant WH6 strains. Laser ablation electrospray ionization-mass spectrometry was used to scan crude culture filtrates for the ion corresponding to 4-formylaminooxyvinylglycine (FVG). Each duplicate well of a 96-well plate contained crude culture filtrate from wild-type *Pseudomonas fluorescens* WH6, a null FVG-mutant strain [WH6-30G (Δ*gvgH*) or WH6-31G (Δ*gvgI*)], or non-inoculated filtrate. Data were collected from 20 laser pulses per sample well, and the peak trace corresponds to the extracted ion chromatogram for sodiated FVG, *m/z* /183.0372.

The ability of LAESI-MS to detect FVG in crude culture filtrates of *P*. *fluorescens* WH6 was subsequently examined. In this experiment, culture filtrates from both wild-type and mutant strains were directly analyzed by LAESI-MS in a 96-well plate format. Using a full scan analysis, a peak at *m/z* 183.037, the sodium adduct, was detected in filtrates collected from wild-type WH6 (Fig 2). This peak was not detected in a media-only control or in culture filtrates from the mutant strains previously shown to display a null-FVG phenotype [16], WH6-30G (Δ*gvgH)* and WH6-31G (Δ*gvgI)* (Fig 3). These results collectively indicate that FVG can be specifically detected in complex samples that have been minimally processed.

The sensitivity of LAESI-MS for detection of FVG was investigated by analyzing a dilution series of culture filtrate from WH6 (2X, 1X, 0.30X, 0.10X, 0.03X and 0.01X) by LAESI-MS. These analyses were performed in selected-ion monitoring mode with scans centered on the sodium adduct of FVG as above. FVG was consistently detected by LAESI-MS in dilutions of filtrates to 0.30X (Table 2). While less sensitive than the bioassays, this method is sufficient to detect FVG in bacterial strains that produce FVG. Under the current instrument settings, the signal intensity is not linear with FVG concentration (Table 2). Development of a quantitative assay would require further optimization, such as including an internal standard, adjusting for detection of FVG in both the high and low range, and addressing potential causes of well-to-well variability.

**Table 2:** Results of biological assays and LAESI-MS detection of FVG for a dilution series of culture filtrate from wild-type *Pseudomonas fluorescens* WH6.

Dilution	Germination Arrest score (SD)^a^	Zone of inhibition, cm^2^ (SD)^b^	LAESI-MS, Signal Intensity (SD)^c^	LAESI-MS, Percent of WT WH6 global avg^d^
2 X	N/A	20.0 (0.1)	1110 (536)	114
1.00 X	1.0 (0.0)	16.1 (0.3)	1027 (455)	106
0.30 X	1.0 (0.0)	12.1 (0.8)	263 (62)	27
0.10 X	1.2 (0.1)	9.0 (0.7)	0 (0)	0
0.03 X	2.1 (0.1)	5.2 (0.2)	4 (5)	0
0.01 X	N/A	0.0 (0.0)	0 (0)	0

^a^ Semi-quantitative germination arrest values are mean of three replicates with SD in parentheses. A score of 4.0 corresponds to normal germination and 0.0 to fully-arrested germination.

^b^ The zone of inhibition of *Erwinia amylovora* in the agar-diffusion assay is shown as the mean of measurements from three agar plates. SD is shown in parentheses.

^c^ LAESI-MS values are shown as the average scan signal intensity centered on the sodium adduct of FVG (*m/z* 183.037) across triplicate wells. SD is shown in parentheses.

^d^ LAESI-MS average scan signal intensity centered on the sodium adduct of FVG (*m/z* 183.037) across triplicate wells, shown as a percentage of the global average for all measurements of WT WH6 filtrate (972, N = 18).

### Detection of FVG in *Pantoea ananatis* BRT175 by LAESI-MS

*Pantoea ananatis* BRT175 is predicted to produce FVG based on the similarity of its PNP-1 gene cluster to the *gvg* cluster of *P*. *fluorescens* WH6, and due to the common activity of their products (Fig 1) [18,19]. However, analyses of extracted culture filtrate from *Pantoea ananatis* BRT175 with TLC were ambiguous because several ninhydrin-reactive compounds co-migrated, and obscured the FVG-associated band (S3 Fig). Despite several modifications to our existing protocol (data not shown), it was not possible to distinguish a FVG-associated band from overlapping bands in the chromatogram. Therefore, we analyzed crude culture filtrates from *Pantoea ananatis* BRT175 by LAESI-MS to determine if *P*. *ananatis* BRT175 produces FVG and if that production requires the PNP-1 cluster. Culture filtrates from wild-type BRT175, wild-type WH6 and strains with mutations in the carbamoyltransferase-encoding gene (*pnp1D*::Tn*5* and Δ*gvgF*) were assayed by LAESI-MS in selected-ion monitoring mode with scans centered on the sodium adduct of FVG. Analysis of the spectra indicated that FVG was detected in the filtrates from both wild-type strains but not in the mutant strains (Table 3). These results are consistent with biological assays performed on these filtrates (Table 3), and confirm both that BRT175 produces FVG and that production of FVG is linked to the PNP-1 cluster.

**Table 3:** Results of biological assays and LAESI-MS analysis for detection of FVG in wild-type and mutant strains of *Pseudomonas fluorescens* WH6 and *Pantoea ananatis* BRT175.

Strain	Genotype	Germination Arrest score at 0.3X (SD)^a^	Zone of inhibition, cm^2^ (SD)^b^	LAESI-MS, Percent of WT WH6 global avg^c^
WT WH6	WT	1.0 (0.0)	16.1 (0.3)	106
WH6-28G	Δ*gvgF*	4.0 (0.0)	0.0 (0.0)	0
WT BRT175	WT	1.0 (0.0)	15.7 (0.2)	43
BRT175-*pnp1D*::Tn*5*	*pnp1D*::Tn*5*	4.0 (0.0)	0.0 (0.0)	1

^a^ The germination arrest score of a 0.3X dilution of culture filtrate is shown. Values are mean score of three replicates with SD in parentheses. In this semi-quantitative assay, a value of 4.0 indicates complete germination and a value of 1.0 indicates that germination is fully arrested.

^b^ The zone of inhibition of *Erwinia amylovora* in the agar diffusion assay is shown as the mean of measurements from three agar plates. SD is shown in parentheses.

^c^ LAESI-MS average scan signal intensity centered on the sodium adduct of FVG (*m/z* 183.037) across triplicate wells, shown as a percentage of the global average for all measurements of WT WH6 filtrate (972, N = 18).

The capability of BRT175 to produce FVG is unusual within its genus. Walterson et al. conducted a PCR-based survey of 117 strains of *Pantoea* and failed to detect *pnp* orthologs in any strain other than BRT175 [18]. Similarly, of the 81 sequenced *Pantoea* strains in the IMG database of the Joint Genome Institute (as of September 2016), no others had orthologous gene clusters. As noted previously [18], the genome of *Pantoea agglomerans* Eh318 does contain homologs of a few of the genes within the *gvg* cluster. However, that strain does not contain homologs of all the genes required for production of FVG, and thus would not be expected to produce FVG.

Based on sequence comparisons, we predict that there are differences in the regulation, transport, and metabolism of FVG in BRT175 compared to WH6. For example, BRT175 lacks orthologs of the ECF sigma factor/anti-sigma factor pair *prtIR*, which have been shown to regulate FVG production in WH6 [23]. FVG production in BRT175 may instead be regulated through other networks. Similarly, export of FVG may also differ, as BRT175 contains only one of the LysE family exporters known to export FVG in WH6 (the ortholog of *gvgJ*). Simultaneous mutation of both *gvgJ* and *gvgK* is lethal in WH6 [16]. Mutation of *pnp1G* in BRT175 may also be lethal, as it was the only gene in the PNP-1 cluster in which a Tn*5* insertion was not recovered [18]. In addition, the PNP-1 cluster of BRT175 lacks orthologs of *gvgD* and *gvgE*, which encode an amidinotransferase and LysE family exporter, respectively. Although *gvgD* and *gvgE* are not required for production of FVG in WH6, they are likely involved in the synthesis of an additional product [16] which would not be produced by BRT175. Additional comparative genomic analyses between BRT175 and WH6 may identify other similarities and differences between these bacteria to shed light on oxyvinylglycine production, regulation, and metabolism. The use of LAESI-MS will facilitate that effort and other on-going studies.

## Conclusions

In the current study, we demonstrate that LAESI-MS can be used to detect FVG in both extracted and crude culture filtrate and differentiates between samples known to contain FVG from those that lack it. Overall, LAESI-MS based detection of FVG is more rapid and requires substantially smaller sample volumes than previous methodologies, enabling potential use in high-throughput analyses. However, detection of relatively low amounts or quantitative analysis of FVG will require further optimization. We demonstrate the utility of this technique by successfully identifying FVG in crude culture filtrates from *Pantoea ananatis* BRT175, the first example of FVG production by a non-pseudomonad.

## Supporting information

S1 Fig Visual key to germination-arrest scores for annual bluegrass seeds. In this semi-quantitative assay, complete germination corresponds to a score of 4.0 and complete arrest corresponds to a score of 0, as described in Banowetz [6]. Figure adapted from Okrent *et al*. (2017).
(PDF)

S2 Fig MS/MS spectrum of fragments from precursor ion *m/z* 183.037 [M+Na] of 4-formylaminooxyvinylglycine (FVG). Peaks noted with asterisks correspond to known fragments from the low resolution ESI-MS/MS spectrum from purified FVG [5].
(PDF)

S3 Fig Thin layer chromatograms showing separation of metabolites in extracted culture filtrates from *Pseudomonas fluorescens* WH6 and *Pantoea ananatis* BRT175. Culture filtrates were extracted with 85% ethanol, and extracts were applied to silica chromatographic plate, and visualized by staining with ninhydrin.
(PDF)
