# Supplementary material for: Detection of 4-formylaminooxyvinylglycine in culture filtrates of Pseudomonas fluorescens WH6 and Pantoea ananatis BRT175 by laser ablation electrospray ionization-mass spectrometry
Source: PLoS One. 2018 Jul 10;13(7):e0200481. doi: 10.1371/journal.pone.0200481 (PMC6039020; doi:10.1371/journal.pone.0200481)
Supplement: S1 Fig — In this semi-quantitative assay, complete germination corresponds to a score of 4.0 and complete arrest corresponds to a score of 0, as described in Banowetz [6]. Figure adapted from Okrent et al. (2017). (PDF) [file pone.0200481.s001.pdf]

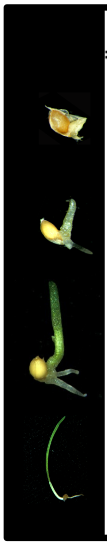

Score	Description
1	The plumule has emerged, but is chlorotic and shorter than the length of the seed. The adventitious root may be visible, but root has not elongated.
2	The plumule length is about equal to that of the seed. The first true leaf has not yet emerged. The roots have elongated.
3	The first true leaf has emerged from, but is shorter than, the coleptile. Roots are elongated.
4	The first true leaf is longer than the coleptile and the roots are elongated.

Figure S1. Visual key to germination-arrest scores for annual bluegrass seeds. In this semi-quantitative assay, complete germination corresponds to a score of 4.0 and complete arrest corresponds to a score of 0, as described in Banowetz [6]. Figure adapted from Okrent *et al.* (2017).
