# Supplementary material for: Detection of 4-formylaminooxyvinylglycine in culture filtrates of Pseudomonas fluorescens WH6 and Pantoea ananatis BRT175 by laser ablation electrospray ionization-mass spectrometry
Source: PLoS One. 2018 Jul 10;13(7):e0200481. doi: 10.1371/journal.pone.0200481 (PMC6039020; doi:10.1371/journal.pone.0200481)
Supplement: S2 Fig — Peaks noted with asterisks correspond to known fragments from the low resolution ESI-MS/MS spectrum from purified FVG [5]. (PDF) [file pone.0200481.s002.pdf]

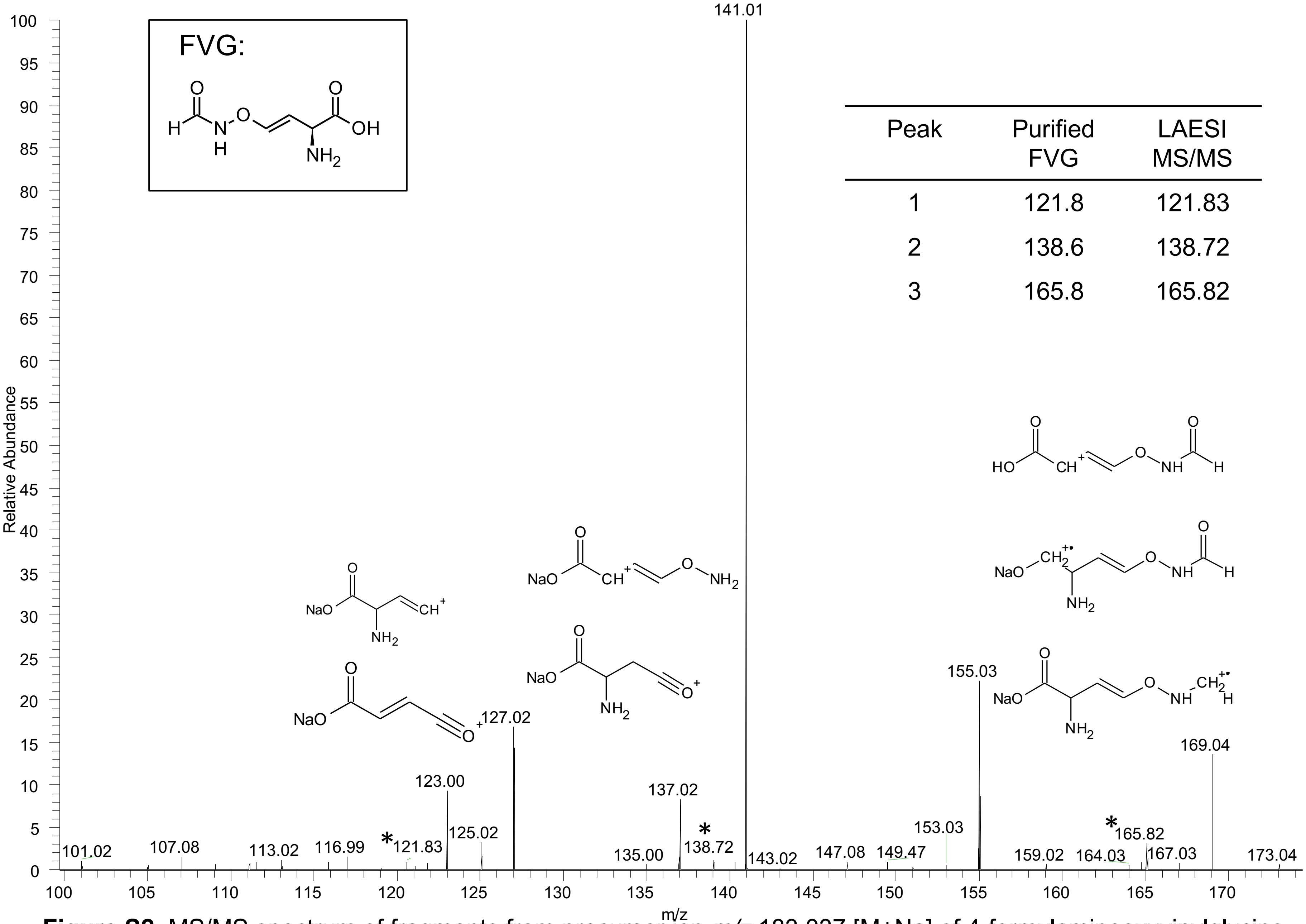

Peak	Purified FVG	LAESI MS/MS
1	121.8	121.83
2	138.6	138.72
3	165.8	165.82

Figure S2. MS/MS spectrum of fragments from precursor ion *m/z* 183.037 [M+Na] of 4-formylaminooxyvinylglycine (FVG). Peaks noted with asterisks correspond to known fragments from the low resolution ESI-MS/MS spectrum from purified FVG [5].
