# Supplementary figures and images for: Detection of 4-formylaminooxyvinylglycine in culture filtrates of Pseudomonas fluorescens WH6 and Pantoea ananatis BRT175 by laser ablation electrospray ionization-mass spectrometry
Source: PLoS One. 2018 Jul 10;13(7):e0200481. doi: 10.1371/journal.pone.0200481 (PMC6039020; doi:10.1371/journal.pone.0200481)

**BRT175**

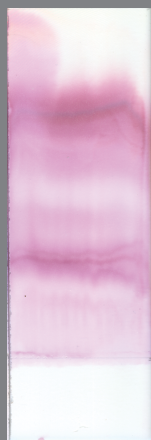

**WH6**

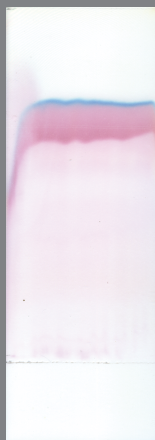

Supplement: S3 Fig — Culture filtrates were extracted with 85% ethanol, and extracts were applied to silica chromatographic plate, and visualized by staining with ninhydrin. (PDF) [file pone.0200481.s003.pdf]
